# Applications of Nanomaterials in Environmental Monitoring and Water Treatment

**DOI:** 10.3390/nano15060426

**Published:** 2025-03-11

**Authors:** Xiangyu Wang, Umer Hayat, Xing Chen

**Affiliations:** 1 School of Resources and Environmental Engineering, Hefei University of Technology, Hefei 230009, Chinaumermughal0526@gmail.com (U.H.); 2 Anhui Provincial Academy of Eco-Environmental Science Research, Hefei 230061, China; 3 National Centre for Water Quality Research, National Institute of Fundamental Studies, Kandy 20000, Sri Lanka

In 2022, 2.2 billion people still lacked access to safely managed drinking water services [1]. Providing clean water safely and affordably is one of the greatest challenges of the 21st century. To address the contamination of water resources with different heavy metals, organic pollutants, and microorganisms, advanced solutions for water decontamination must be found [2,3]. Nanotechnology is an innovative and advanced technology that can be used to manipulate this issue on the nanoscale, providing new techniques for environmental monitoring and water treatment processes. Nanomaterials are more efficient because of their high surface areas, volume ratios, reactivity with pollutants, and tunability for the eradication of contaminants from water. This Editorial explores our current knowledge of nanomaterials and how they are being used in water treatment to remove contaminants from water and wastewater [2,4].

Ceroni et al. prepared a synthetic form of multi-walled carbon nanotubes (MWCNT-S) containing benzenesulfonate. They were synthesized and analyzed using various characterization techniques. This variation significantly enhanced the nanotubes’ ability to dissolve in water and improved their adsorption competence for methylene blue (MB) associated with unmodified MWCNT-S and native activated carbon materials [3].

Wang et al. developed activated carbon fiber felt and modified it with a MgO nanosheet (MgO@ACFF) for the removal of fluoride. This method allowed more than 90% of the tested fluoride to be removed within 100 min. MgO@ACFF adsorbed 212.2 mg/g of fluoride at neutral pH and effectively removed fluoride from water in a wide pH (2–10) range, an excellent result for this application [5].

El-Ghobashy et al. developed a NiO/polydopamine (NiO/PDA) nanocomposite to effectively remove Methyl violet 2B dye from wastewater. The material was exposed to particles with an average size of 18 nm and a surface area of 110.591 m^2^/g. The NiO/PDA nanocomposite exhibited a more sophisticated dye adsorption capacity (284 mg/g), associated with bare NiO (126 mg/g). The complex exhibited excellent reusability, maintaining stability across three adsorption–regeneration cycles [6].

Matos et al. manufactured ZnO nanocrystals in three separate forms using latex from Brosimum parinarioides (bitter Amapá) and Parahancornia amapa (sweet Amapá) as chelating agents. The nanocrystals, recognized into pitanga-like, teetotum-like, and cambuci-like forms, were characterized as an unpolluted hexagonal wurtzite phase. The teetotum-like ZnO revealed the best photocatalytic performance, achieving 85% MB dye removal, ascribed to its inimitable form and oxygen flaws. The results of this study show that these latex-derived ZnO nanocrystals have promising photocatalytic activity and can be manufactured effectively using latex from plants from the Amazon rainforest [7].

Liu et al. investigated the degradation of sulfamethoxazole (SMX) using a photo-Fenton catalyst composed of hydroxyl-modified UIO-66 (HO-UIO-66) dispersed in diatomite. Initially, the catalyst achieved 94.7% SMX degradation; however, to improve stability, a calcination step at 300 °C was required. The resulting composite (HO-UIO-66/DE-300) exhibited slightly reduced efficiency (93.8%), while ESR data identified h^+^, •OH, •O_2_^−^, and ^1^O_2_ as key reactive species [8].

Although nanomaterials have been extensively utilized in environmental monitoring and water treatment, there is still a necessity to develop novel nanotechnologies for practical and emerging water issues. For instance, atomically dispersed Co-Mn dual-site catalysts (Co-Mn/CN DACs) exhibit low metal-ion leaching and allow efficient PMS activation for the elimination of petroleum hydrocarbon contamination in water [9]. An optical strontium-specific nanosensor with ultrahigh binding affinity has been developed. It offers an ultra-low detection limit of 0.5 nM, a wide sensing range of eight orders of magnitude, a rapid response of less than 10 s, and high selectivity against 31 common ions [10]. Most importantly, the integration of artificial intelligence (AI) into materials science presents transformative potential, particularly in accelerating nanomaterial discovery through the machine learning-assisted optimization of synthesis parameters, environmental response patterns, and economic feasibility factors [11]. This data-driven strategy facilitates the rational design of engineered functional nanomaterials, effectively overcoming the inherent limitations of conventional trial-and-error screening methodologies. In summary, it is only through the synergistic integration of technological innovation and practical implementation that nanomaterials can evolve into a sustainable solution for environmental remediation.

## References

[1] WHO (2023). Progress on Household Drinking Water, Sanitation and Hygiene 2000–2022: Special Focus on Gender.

[2] Al-Amrani W.A., Onaizi S.A. (2024). Adsorptive removal of heavy metals from wastewater using emerging nanostructured materials: A state-of-the-art review. Sep. Purif. Technol..

[3] Ceroni L., Benazzato S., Pressi S., Calvillo L., Marotta E., Menna E. (2024). Enhanced adsorption of methylene blue dye on functionalized multi-walled carbon nanotubes. Nanomaterials.

[4] Islam M.T., Al Mamun M.A., Halim A.F.M.F., Peila R., Sanchez Ramirez D.O. (2024). Current trends in textile wastewater treatment—Bibliometric review. Environ. Sci. Pollut. Res..

[5] Wang D.-C., Xu M.-D., Jin Z., Xiao Y.-F., Chao Y., Li J., Chen S.-H., Ding Y. (2023). Synthesis and characterization of porous MgO nanosheet-modified activated Carbon Fiber Felt for Fluoride Adsorption. Nanomaterials.

[6] El-Ghobashy M.A., Hashim H., Darwish M.A., Khandaker M.U., Sulieman A., Tamam N., Trukhanov S.V., Trukhanov A.V., Salem M.A. (2022). Eco-friendly NiO/polydopamine nanocomposite for efficient removal of dyes from wastewater. Nanomaterials.

[7] Matos R.S., Attah-Baah J.M., Monteiro M.D., Costa B.F., Mâcedo M.A., Da Paz S.P., Angélica R.S., de Souza T.M., Ţălu Ş., Oliveira R.M. (2022). Evaluation of the photocatalytic activity of distinctive-shaped ZnO nanocrystals synthesized using latex of different plants native to the Amazon rainforest. Nanomaterials.

[8] Liu H.-L., Zhang Y., Lv X.-X., Cui M.-S., Cui K.-P., Dai Z.-L., Wang B., Weerasooriya R., Chen X. (2023). Efficient Degradation of Sulfamethoxazole by Diatomite-Supported Hydroxyl-Modified UIO-66 Photocatalyst after Calcination. Nanomaterials.

[9] Guo Q., Yu X.-F., Zhang K., Xia L., Liu S., Zhang W., Du Y., Tang H., Peng Y., Li Z. (2024). Atomically dispersed Co-Mn dual sites anchored in photoresponsive carbon nitride mediated peroxymonosulfate activation for elimination of petroleum hydrocarbon in water. Appl. Catal. B Environ..

[10] Du X.F., Xie H., Qin T.Y., Yuan Y.H., Wang N. (2024). Ultrasensitive optical detection of strontium ions by specific nanosensor with ultrahigh binding affinity. Nat. Commun..

[11] Ding Z., Liu S., Lv X., Duan C., Liu F., Liu R., Chen X. (2025). Application of machine learning strategies in screening transition metal oxide based ozonation catalysts for BAA degradation. J. Water Process Eng..

